# Editorial: Deep learning for high-dimensional sense, non-linear signal processing and intelligent diagnosis, vol II

**DOI:** 10.3389/fpsyt.2026.1794454

**Published:** 2026-03-03

**Authors:** Hengjin Ke, Chang Cai, Lihua Yao, Dan Chen

**Affiliations:** 1 School of Computer, Hubei Polytechnic University, Huangshi, China; 2 Faculty of Artificial Intelligence Education, Central China Normal University, Wuhan, China; 3 Department of Medical Social Sciences, Feinberg School of Medicine, Northwestern University, Chicago, IL, United States; 4 School of Computer Science, Wuhan University, Wuhan, China

**Keywords:** classification, deep learning, EEG, interpretation, non linear

## 1 Introduction

Psychiatry stands at a pivotal turning point shaped by rapid technological advances and pressing clinical demands (1). Mental health disorders, defined by multifaceted etiologies and heterogeneous presentations, have traditionally relied on subjective assessments and qualitative interviews. However, the advent of high-dimensional neuroimaging and electrophysiological data—such as EEG and MRI—offers an opportunity to move toward objective, biomarker-based diagnostics. These modalities capture the brain’s dynamic, non-linear, and spatially distributed activity, yet analyzing such data remains challenging due to noise, high dimensionality, and inter-individual variability.

Deep learning has demonstrated exceptional potential across biomedical domains, including neuroimaging and genomics, by automatically learning hierarchical representations from raw or minimally processed data. Its capacity to model complex, non-linear patterns makes it particularly suitable for deciphering psychiatric signals. This computational capability enables researchers to transcend traditional linear methods and uncover subtle biomarkers underlying mental disorders.

**Figure 1:**
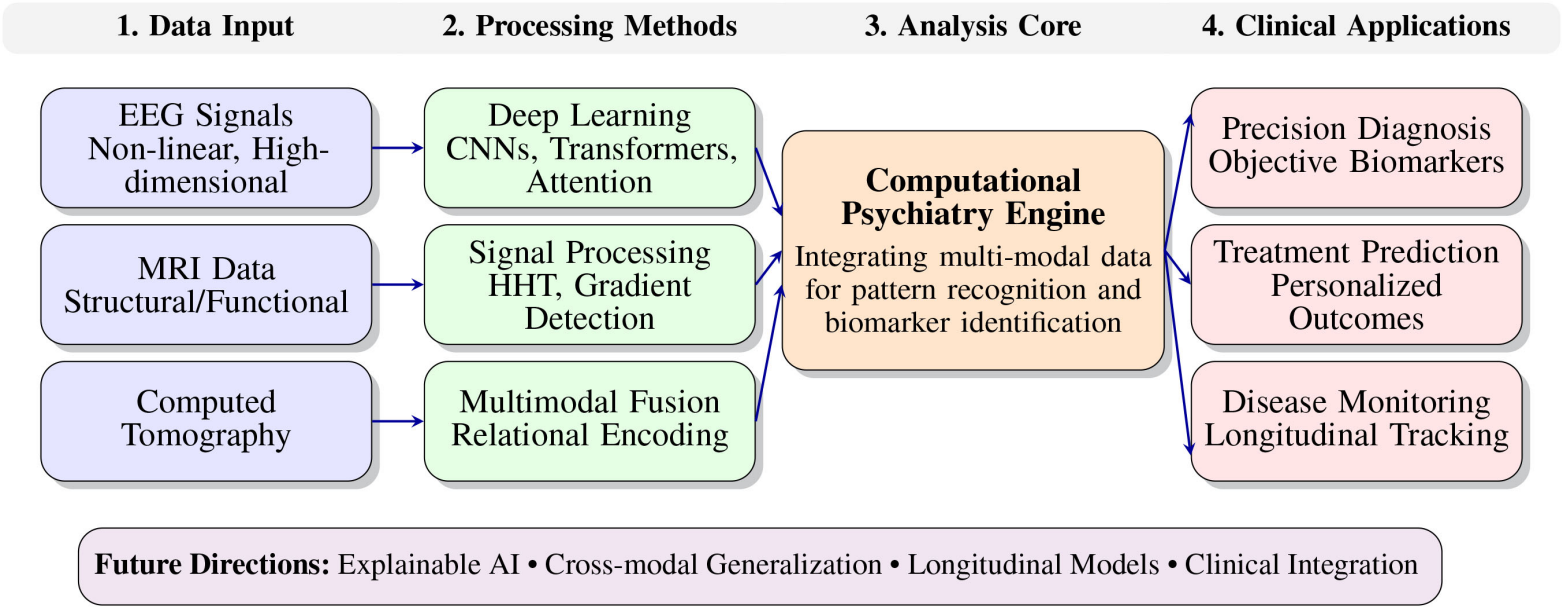
The integration of deep learning and high-dimensional signal processing in computational psychiatry. The four-stage workflow illustrates the transformation from multimodal data inputs through advanced analytical techniques to clinically relevant applications.

To conceptualize this integrative approach, **Figure 1** illustrates a four-stage workflow that encapsulates the synergy between deep learning and high-dimensional signal processing in computational psychiatry. The pipeline begins with multimodal data inputs [EEG (2), MRI (3), Computed Tomography (4)], progresses through advanced analytical methods (deep learning architectures, signal processing techniques, multimodal fusion), and culminates in clinically relevant applications such as precision diagnosis, treatment prediction (5), and longitudinal monitoring. This structured framework highlights how computational intelligence (6) can transform raw data into actionable clinical insights, thereby bridging the gap between algorithmic innovation and psychiatric practice.

As the second volume in our series, this Research Topic consolidates cutting-edge work at the intersection of deep learning and high-dimensional signal processing in psychiatry. By highlighting methods that enhance interpretability, accuracy, and robustness in computational diagnostics, we aim to accelerate innovation and foster collaboration between computational scientists and clinicians, ultimately translating algorithmic progress into practical tools for mental healthcare.

## 2 Objectives of the Research Topic

This Research Topic seeks to advance computational psychiatry through innovations that integrate deep learning with advanced signal processing. The overarching objective is to move beyond conventional analytical methods and develop robust, interpretable, and clinically actionable tools capable of deciphering the complexity of psychiatric data for improved diagnosis and personalized treatment prediction.

The featured studies exemplify this effort through diverse approaches: predicting Cognitive Behavioral Therapy efficacy in post-stroke depression using interpretable machine learning (Lin and Yu; detecting depression via attention-based multi-scale convolutional networks on EEG (Yan et al.; screening Major Depressive Disorder by embedding the Hilbert-Huang Transform within a self-attention neural network (Chen et al.; designing a Sobel Neural Network that learns gradient-based features directly from EEG topographies (Yang and Ye; and developing a multimodal framework for intelligent nonlinear signal interpretation (Pan). Collectively, these studies illustrate the critical value of integrating domain-specific signal processing techniques—such as time-frequency decomposition, spatial gradient detection, and multimodal fusion—into deep learning architectures. This synergy is essential for managing the non-linear, non-stationary, and noisy characteristics of neurophysiological data, thereby enabling more precise and reliable biomarkers for mental health disorders.

## 3 Summaries of key contributions

### 3.1 Machine learning for treatment prediction in post-stroke depression

Lin and Yu conducted a retrospective study evaluating the efficacy of Cognitive Behavioral Therapy (CBT) for post-stroke depression (PSD) and developed an interpretable machine learning model to predict individual treatment responses. Their random forest classifier achieved an AUC of 0.897, identifying baseline depression severity (PHQ-9), self-efficacy, and social support as key predictors of CBT success. This study marks a step toward personalized, predictive rehabilitation strategies, offering a clinically applicable tool for optimizing psychological interventions in stroke survivors.

### 3.2 Attention-based deep learning for EEG depression detection

Yan et al. propose AMCCBDep, an end-to-end hybrid model that integrates Attention-based Multi-scale Parallel Convolution (AMPC), Conformer, and BiGRU for EEG-based depression (7) recognition. Achieving an accuracy of 98.68% within the MODMA dataset evaluation, the model demonstrates robustness even with fewer electrodes. Through channel attention and temporal-frequency learning, the study advances scalable, high-accuracy EEG diagnostics suitable for real-world clinical use.

### 3.3 Hilbert-Huang transform embedded self-attention network for EEG classification

Chen et al. introduce a framework embedding the Hilbert-Huang Transform (HHT) into a self-attention neural network for Major Depressive Disorder (MDD) screening. The HHT layer adaptively decomposes EEG signals into intrinsic mode functions, capturing the non-linear and non-stationary dynamics of depression. With an accuracy of 98.78% on the employed dataset, the model outperforms conventional approaches while providing interpretable time-frequency representations, demonstrating how traditional signal processing can enhance deep learning-based neuropsychiatric analysis.

### 3.4 Sobel neural network for EEG-based depression screening

Yang and Ye present a Sobel Network that intrinsically incorporates gradient-based operations into convolutional layers for EEG-based depression detection. Unlike standard preprocessing pipelines, this end-to-end architecture emphasizes edge-like spatial gradients in EEG topographies—features closely associated with depression-related disruptions in neural connectivity. The network achieves 98.67% accuracy under the experimental conditions reported, showcasing a principled integration of image-processing priors with deep learning for neurodiagnostic applications.

### 3.5 Multimodal deep learning for nonlinear signal interpretation

Pan addresses the challenge of integrating heterogeneous data by proposing a multimodal deep learning framework. It features a Fusion-Aware Relational Encoder (FARE) to model high-order interactions between modalities like EEG and MRI, and a Modality-Aligned Optimization Strategy (MAOS) to ensure balanced learning. This approach advances comprehensive psychiatric assessment by effectively combining multiple data sources, directly tackling the multimodal fusion challenge critical for future research.

## 4 Discussion

The contributions in this volume, while promising, must be scrutinized within the broader landscape of computational psychiatry. Recent methodological innovations, such as deep wavelet self-attention non-negative tensor factorization (8) and deep wavelet temporal-frequency attention (9), represent significant strides in modeling non-linear fMRI dynamics, yet they also reveal a persistent tension between model complexity and interpretability. While these approaches offer enhanced discriminability for conditions like autism spectrum disorder and depression (10, 11), their clinical translation remains hampered by a lack of standardized validation across diverse cohorts and imaging protocols. Furthermore, the reliance on high-quality, labeled data poses a critical bottleneck; generative adversarial networks coupled with tensor decomposition (10) attempt to mitigate data scarcity, but such synthetic data may inadvertently amplify biases or obscure biologically plausible variations.

Beyond fMRI, EEG-based frameworks have made commendable progress in scalability and real-time applicability (12). However, the pursuit of ever-higher accuracy (often exceeding 98%) risks creating an illusion of perfection, masking underlying issues of generalizability, demographic bias, and sensor variability. The field must therefore pivot from isolated performance metrics toward robust, ethically aligned deployment—a shift that demands closer collaboration between computational researchers, clinicians, and ethicists.

Ultimately, the true measure of success for computational psychiatry (13) lies not in algorithmic sophistication alone, but in its capacity to generate clinically actionable, interpretable, and equitable insights. Future work must prioritize transparent model architectures, rigorous external validation, and integrative frameworks that bridge the gap between high-dimensional data and holistic patient narratives.

## 5 Conclusion and future directions

Collectively, the studies in this volume underscore the transformative potential of deep learning and advanced signal processing in modern psychiatry. From predicting therapeutic outcomes to decoding complex neurophysiological signals and integrating multimodal data, these contributions chart a clear course toward more precise, personalized, and data-informed mental healthcare. However, as highlighted in the Discussion, several critical challenges must be addressed to realize this vision.

Future research should focus on:

- Enhancing generalizability and robustness: Developing models that maintain consistent performance across populations, clinical sites, and acquisition protocols, moving beyond single-dataset benchmarks.
- Multimodal fusion architectures: Combining EEG, MRI, digital phenotyping, and clinical records to capture the multidimensional complexity of psychiatric disorders, while preserving interpretability.
- Federated and privacy-preserving learning: Enabling multi-institutional model training without compromising data confidentiality, fostering collaborative and inclusive research ecosystems.
- Explainable AI frameworks: Bridging model predictions with clinical reasoning by offering interpretable, transparent insights that align with neurobiological and psychological constructs.
- Longitudinal modeling: Tracking disease trajectories and treatment responses over time to enable dynamic mental health forecasting and early intervention.
- Ethical and equitable AI: Actively addressing biases in data and algorithms to ensure that computational tools benefit diverse populations without perpetuating disparities.

We hope this volume inspires continued innovation at the intersection of computational science and psychiatry, ultimately advancing a future of more accessible, accurate, and compassionate mental healthcare worldwide.
